# Audit and feedback with or without training in-practice targeting antibiotic prescribing (TiPTAP): a study protocol of a cluster randomised trial in dental primary care

**DOI:** 10.1186/s13012-021-01098-z

**Published:** 2021-03-30

**Authors:** Beatriz Goulao, Claire Scott, Irene Black, Jan Clarkson, Lee McArthur, Craig Ramsay, Linda Young, Eilidh Duncan

**Affiliations:** 1 Health Services Research Unit, University of Aberdeen, Aberdeen, Scotland; 2 NHS Education for Scotland, Edinburgh, UK; 3 Dental Health Services Research Unit, Dundee Dental School, The University of Dundee, Dundee, UK

**Keywords:** Audit and feedback, Cluster randomised trial, Quality improvement, Implementation, Antibiotic prescription

## Abstract

**Background:**

Antimicrobial resistance is an increasingly serious threat to global public health and patient safety. Overuse of antibiotics has aggravated this issue. Around 7% of all antibiotics in Scotland are prescribed by dentists. Audit and feedback has been shown to decrease these prescriptions, but there is evidence that dentists still prescribe unnecessarily. Our aim is to compare the effectiveness of a theory-informed in-practice training session (TiPTAP) in addition to individualised audit and feedback, with audit and feedback alone for reducing antibiotic prescribing by NHS dentists working in NHS primary care dental practices.

**Methods:**

We will conduct a 2-arm parallel cluster randomised trial: out of 228 practices, 114 will be randomised to the theory-informed in-practice training session targeting antibiotic prescribing and individualised audit and feedback; 114 practices will be randomised to audit and feedback alone. The theory-informed session will include (a) an introductory session including several behaviour change techniques; (b) problem solving discussion, setting and recording action plans; (c) practice-level prescribing feedback discussion. The primary outcome is the number of antibiotic items per 100 NHS treatment claims over a 1-year period post-randomisation for each dentist. Secondary outcomes are the number of amoxicillin 3 g and broad spectrum antibiotics prescribed per 100 NHS treatment claims over a 1-year period; amoxicillin 3 g and broad spectrum antibiotics defined daily doses of antibiotics per 100 claims. Process measures include fidelity, knowledge, and confidence. Primary and secondary outcomes will be obtained using routine data.

**Discussion:**

This study provides the opportunity to robustly assess the effect of adding an in-practice training co-intervention to audit and feedback. Its behaviour change theory-informed content will allow replication of the different components and can inform future training interventions.

**Trial registration:**

ISRCTN, ISRCTN12345678. Registered 18 June 2020.

Contributions to the field

- Antibiotic resistance is a serious threat to global public health. Overuse of antibiotics has aggravated this issue. Around 7% of all antibiotics dispensed in Scotland are prescribed by dentists and there is evidence that a significant proportion is prescribed unnecessarily. The COVID-19 pandemic aggravated the problem due to the need for remote management of disease.
- Audit and feedback (A&F), a summary of clinical performance provided over a specific period, is an effective intervention at changing healthcare professional behaviours. However, its effectiveness varies considerably, and further work needs to be done to understand how to maximise its impact. A potential way forward is to assess A&F effectiveness alongside a co-intervention. In-practice training has been shown to work in decreasing prescriptions, but it is poorly reported and difficult to replicate.
- Our work aims to compare the effectiveness of individual A&F with in-practice training compared with individual A&F alone in primary care National Health System dental practices in Scotland. Use of behaviour change theory in the in-practice training will ensure replicability ofthe active ingredientes of the in-practice training. The results will inform the implementation science methodology community regarding potential ways to maximise A&F’s impact and will contribute to the literature on management of primary care antibiotic prescription.

## Background

Antimicrobial resistance is an increasingly serious threat to global public health and patient safety [1]. Overuse of antimicrobials has resulted in many agents becoming relatively ineffective for simple infections due to emerging bacterial resistance, and reducing the inappropriate use of antibiotics is a key priority of Scottish Government [2].

Approximately, 7% of all antibiotics dispensed in community pharmacies in Scotland are prescribed by dentists [3]. National guidance to support dentists to make appropriate antibiotic prescribing decisions was first published by the Scottish Dental Clinical Effectiveness Programme (SDCEP) in April 2008 and last updated in January 2016 with an accompanying app released in January 2019 [4].

From 2006 to 2012 routine dental prescribing data monitored by NHS Education for Scotland’s (NES’s) Translation Research in a Dental Setting (TRiaDS) programme demonstrated that, despite the SDCEP guidance, the number of antibiotics prescribed by dentists was steadily increasing year-on-year. In recent years, the number of antibiotics prescribed by dentists has demonstrated a downward trend [3]. However, current evidence from NES national antibiotic prescribing audits clearly demonstrates that dentists are still prescribing unnecessarily when there are no clinical indications to do so. The COVID-19 pandemic led to an exacerbation of the issue with increased antibiotic prescriptions by up to 60% when compared to the previous year in England [5]. This was due to restrictions in dental access that resulted in remote management of patients; during this time, dentists were advised to provide advice, analgesics and antibiotics, where appropriate [6]. Similar advice and prescribing trends were observed in Scotland during this period. Therefore, there is a need to support dentists to improve their antibiotic prescribing.

One way that can effectively reduce antibiotic prescribing in dentistry is the provision of Audit and Feedback (A&F) [7]. A&F is a widely used intervention directed at health professionals to improve the implementation of guidance and research findings into clinical practice [8]. A&F is defined as any summary (written, electronic or verbal) of clinical performance of healthcare provided over a specified period [8]. It provides objective data showing discrepancies between current and target performance and can include comparison of individual performance in relation to other health professionals. A&F is evidence-based, scalable and relatively inexpensive contributing to its popularity.

Despite its widespread use, the science around how, when and why A&F works best is still lacking. In the most recent update of the Cochrane review of 140 trials of A&F interventions, median absolute improvement in health professional compliance with desired practice was 4.3%, but the range of effect was highly variable [8]. Given the enduring popularity and established effectiveness of A&F, identifying factors which distinguish between more and less successful A&F interventions is a challenge that requires urgent attention [8, 9].

One way to potentially optimise the effect of A&F is to provide co-interventions. In-practice training (also known as ‘academic detailing’ or ‘outreach education’) is a method of face-to-face training of healthcare professionals that often accompanies A&F. There is some evidence to suggest it may reduce inappropriate prescribing by medical practitioners [10–12]; however, there is considerable variation in how the intervention is described, e.g. “education”, “discuss content of the guidelines and encourage rational use of antibiotics” [10] and in the level of detail provided in the reports of studies investigating the effectiveness. It is therefore likely that the content of academic detailing varies from trial to trial making generalisations about its effectiveness very difficult. Specifying the active ingredients or ‘behaviour change techniques’ of academic detailing ensures more precisely specified content and allows for testing of different components and combinations of components.

### Setting

In Scotland, NHS Education for Scotland’s (NES) Quality Improvement in Practice Training (QIiPT) team delivers Infection Control (IC) training to around 300 general dental practices across Scotland per year. All NHS dental practices are required to receive QIiPT IC training at least once every 3 years in order to meet NHS dental practice inspection requirements. The QIiPT team are expanding their remit by adding a component focusing on antibiotic prescribing to their training package.

### Trial aim and objectives

The aim is to compare the effectiveness of a theory-informed in-practice training session (TiPTAP) in addition to individualised audit and feedback, with audit and feedback alone for reducing antibiotic prescribing by NHS dentists working in NHS primary care dental practices. Audit and feedback alone was selected as the control arm since it has been shown to be effective in reducing antibiotic prescriptions by NHS primary care dentists in Scotland [7] and, therefore, should be the standard intervention.

#### Primary objective

To compare the effectiveness of TiPTAP training plus individualised audit and feedback with individualised audit and feedback alone on dentists’ antibiotic prescribing rates.

#### Secondary objectives

To compare the effectiveness of TiPTAP training plus individualised audit and feedback with individualised audit and feedback alone on:

- The number of amoxicillin 3 g items per 100 claims.
- The number of broad spectrum or “second-line” antibiotic items (clindamycin, co-amoxiclav, clarithromycin, cefalexin and cefradine) per 100 claims.
- Defined daily doses of antibiotics per 100 claims.
- Defined daily doses of amoxicillin 3 g per 100 claims.
- Defined daily doses of broad spectrum or “second-line” antibiotics (clindamycin, co-amoxiclav, clarithromycin, cefalexin and cefradine) per 100 claims.

## Methods

### Study design

The study is a 12-month cluster randomised controlled trial conducted in NHS General Dental Practices across all 14 health boards in Scotland.

### Setting and participants

The trial is set in general dental practices that have booked QIiPT IC training. Training was historically delivered to whole practice teams (including dentists, dental nurses, reception staff and practice managers) at the practice premises by a team of two QIiPT trainers. Currently, and due to the COVID-19 pandemic, training is delivered virtually to the whole practice team.

### Eligibility

**Fig. 1:**
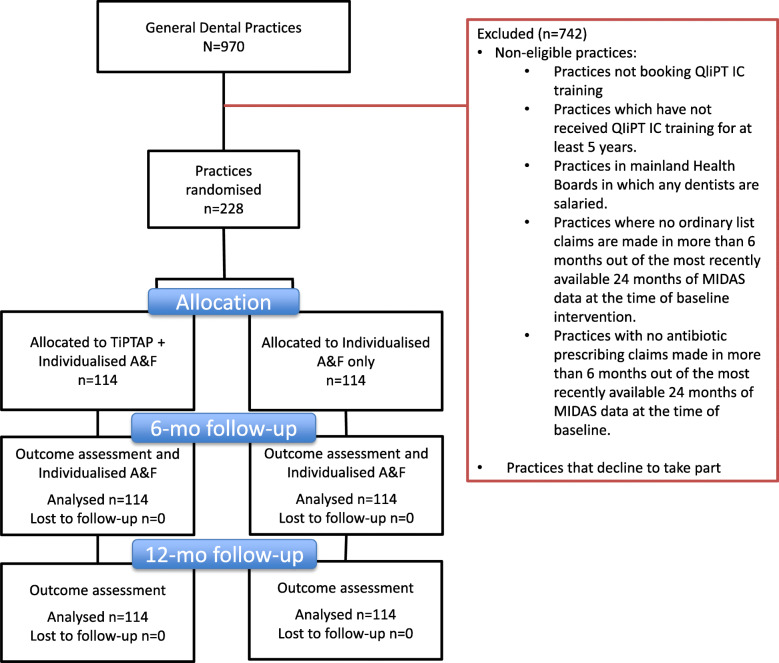
Flow diagram

Dental practice inclusion criteria is available in Fig. 1. Dentists participating in the trial need to be working in a practice which has booked QIiPT IC training and has agreed to take part in TiPTAP; and need to have treatment (claims) data available in the national dataset so audit and feedback can be provided.

### Interventions

#### Intervention group

Practices will receive the following:

i) TiPTAP intervention: QIiPT IC training with the inclusion of a theory-informed in-practice training session (which has been worked into the standard QIiPT IC training and therefore takes no additional time)
ii) Individualised A&F

##### TiPTAP intervention

The QIiPT IC training aims to enable practices to reflect on existing processes and to consider any changes required for improvement and compliance with SDCEP guidance. Its 2-h standardised content includes a PowerPoint presentation, practical demonstration and discussion. Action plans are set with the practice on the day of the visit and followed up after 8 weeks to provide advice and support if the action plans have not been achieved. The topics covered are tailored to the need of the practice and can cover hand hygiene, cleaning instruments, environmental cleaning and the testing and maintenance of decontamination equipment.

The TiPTAP intervention consists of the following:

- An introductory presentation delivered by a QIiPT trainer
- Problem solving discussion and setting and recording action plans.
- Feedback of practice-level antibiotic prescribing data.

The intervention is theory informed and includes precisely specified content and replicable behaviour change techniques (BCTs) [13]. The BCTs included are 1.2 Problem solving, 1.4 Action planning, 1.6 Discrepancy between current behaviour and goal, 2.2 Feedback on behaviour, 4.1 Instruction on how to perform the behaviour, 5.1 Information about health consequences, 5.2 Salience of consequences, 5.3 Information about social and environmental consequences, 5.5 Anticipated regret, 9.1 Credible source, 12.1 Restructuring the physical environment. Table 1 includes a breakdown of the TiPTAP intervention.

**Table 1:** Breakdown of TiPTAP intervention

Intervention component	Mode of delivery	Delivered to	When
Feedback of practice-level and individualised antibiotic prescribing data	Documents sent electronically	Practice contact (practice data) Individual dentists (individual data)	Prior to QIiPT visit
Introductory presentation	Remote online meeting (group)	Practice team	On the QIiPT visit before infection control and decontamination training completed
Problem solving discussion	Remote online meeting (group)	Practice team	On the QIiPT visit day after the introductory educational presentation
Tools 1. Prescribing data plus guidance for use 2. ‘Antibiotics do not cure toothache’ Poster (digital copy) 3. Link to ‘antibiotics do not cure toothache’ patient leaflets 4. SDCEP bacterial infections management flow chart (digital copy) 5. Information relating to delayed prescribing 6. Script for reception staff 7. Any other relevant resources	Provision of list of links to online resources	Practice team	During problem solving discussion on QIiPT visit day
Action plans	Discussed at face to face training visit Completed and returned electronically	Practice team	To be completed and returned within 8 weeks

The problem solving discussion component of the intervention will include discussion about the challenges involved when deciding to prescribe antibiotics or not. Discussion will also involve the presentation of 6 ‘tools’ that practices can use to assist in optimising prescribing efforts. The tools are practice posters and links to leaflets designed to target patient expectations (e.g. ‘antibiotics do not cure toothache’ materials), patient behaviour (patient information and instructions relating to delayed prescribing and post-extraction and emergency treatment guidance) and practice team behaviour (e.g. SDCEP bacterial infections management guidance poster, guidance regarding delayed prescriptions, scripts for negotiating antibiotic use with patients, guidance for reception staff).

QIiPT trainers will discuss action plan setting with the practice and will give an example action plan. Practices will be asked to complete and return a template specifying the area for improvement, goal, plan, who is responsible, how will progress be measured and whether goal has been achieved within 8 weeks of the practice visit.

The BCTs within TiPTAP were chosen for inclusion based on previous research [14], expert consensus (at the TRiaDS Research Methodology Group meeting February 2017), and APEASE criteria (Affordability, Practicability, Effectiveness/cost-effectiveness, Acceptability, Side-effects/safety, Equity [15]) applied by members of the study team (IB, ED and CR). The TiPTAP intervention includes practice-level A&F, with similar features to the dentist-level A&F described below. The practice-level audit and feedback will be sent in advance of the training session and discussed at the time of the TiPTAP intervention delivery.

The feasibility and acceptability of TiPTAP was evaluated in a small number of practices undertaking QIiPT infection control training. Delivery of TiPTAP was found to be feasible and the TiPTAP training intervention was considered acceptable by practice staff and QIiPT trainers.

##### Individualised audit and feedback

**Fig. 2:**
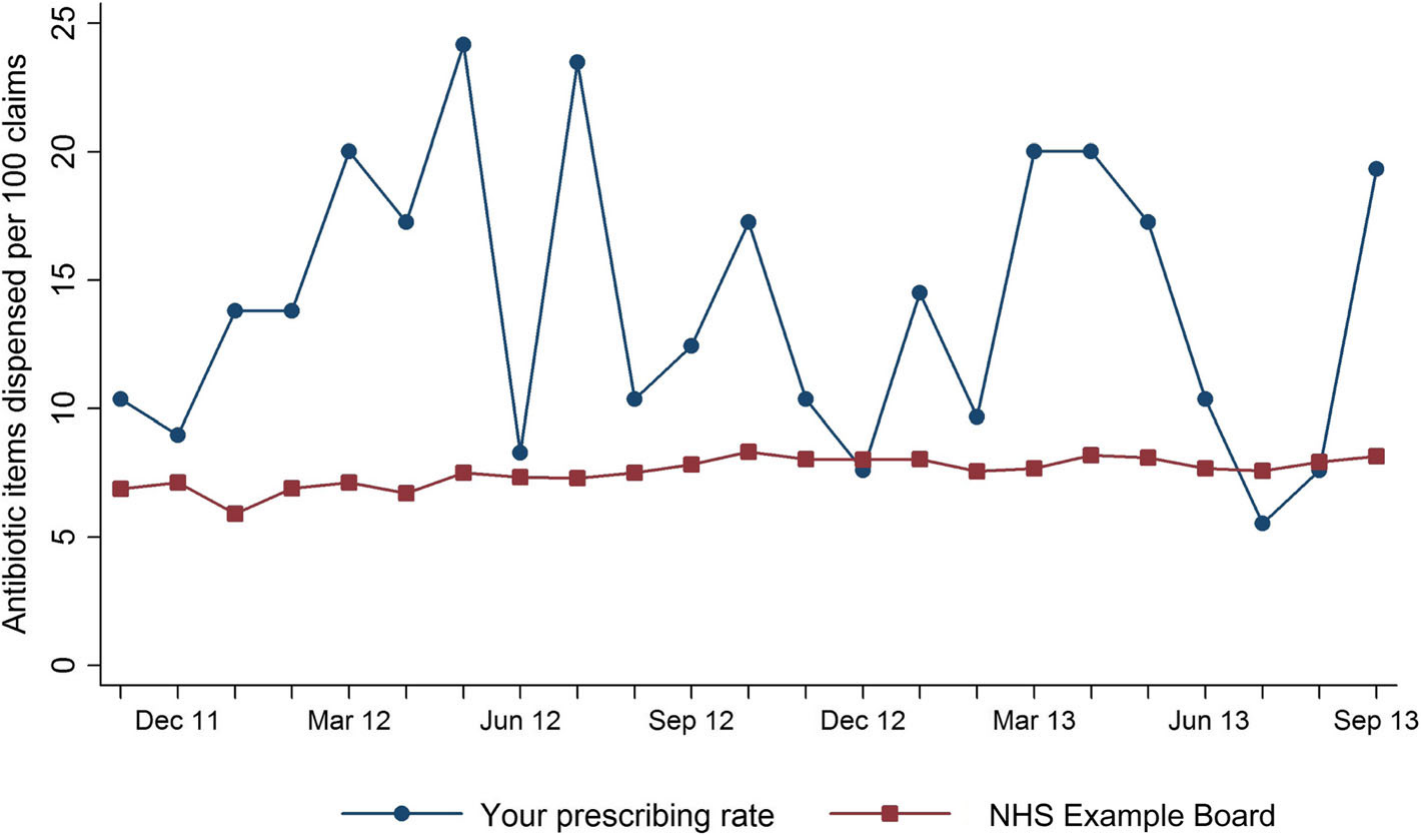
Template for the individualised audit and feedback intervention presenting data on a dentist's antibiotic prescribing over a period of time

The individualised A&F intervention materials will be developed based on the findings of the RAPiD trial [7] which showed that the most effective A&F intervention included a text-based behaviour change message. The monthly number of claims will be determined as the number of ordinary list claims for treatment recorded in the MIDAS database each month (i.e. claims made for NHS treatment carried out for patients registered under a dentist's standard list number at a given location). Claims made on other lists, e.g. emergency, trainer and assistant will be excluded. Monthly rates of antibiotic prescriptions will be presented to each dentist. The prescribing rate is the monthly number of prescription items dispensed divided by the mean monthly number of NHS treatment claims (multiplied by 100). The feedback will contain retrospective prescribing data taken from the previous up to 24 months. Monthly prescribing rates for territorial Health Boards will be calculated similarly based on total antibiotic items prescribed and total number of NHS treatment claims within each territorial Health Board. The individualised A&F will be delivered to all dentists 2 weeks after their training session. An example of individual A&F can be found in Fig. 2.

### Control group

The control group will receive (i) training as usual and (b) individualised audit and feedback.

### Outcomes

The primary outcome is the number of antibiotic items per 100 NHS treatment claims over a 1-year period post-randomisation for each dentist (with specific time points of interest being 6 and 12 months post-randomisation). The secondary outcomes, measured over the same period, are the following:

- Total number of amoxicillin 3 g per 100 NHS treatment claims.
- Total number of broad spectrum or “second-line” antibiotics (clindamycin, co-amoxiclav, clarithromycin, cefalexin and cefradine) per 100 claims.
- Defined daily doses of antibiotics per 100 claims.
- Defined daily doses of amoxicillin 3 g per 100 claims.
- Defined daily doses of broad spectrum or “second-line” antibiotics (clindamycin, co-amoxiclav, clarithromycin, cefalexin and cefradine) per 100 claims.

### Process measures

Fidelity, knowledge and confidence will be assessed via an online individual questionnaire distributed to all in-practice training attendees at the end of the session. This will include a question on attendees’ professional role in the dental team to allow us to describe who attended the training. We will collect whether practices received the intervention as intended via the QIiPT team. Fidelity will be further assessed via an in-depth parallel process evaluation (protocol in preparation).

### Recruitment and randomisation

When practices contact the QIiPT team to book their IC training visit, they will be assessed for eligibility and informed there is a possible new training component in their session. If the practices are interested in the new training component, they will be sent an information sheet and online practice consent form via email. Once the consent forms have been completed, they will be attributed a study number by the trial office in Dundee. The trial statistician, blinded to the practices’ identity, will receive a list of practices with an anonymised identification and randomise them. The allocation will be sent back to the trial office who will unblind the data and communicate it to the QliPT team. The unit of randomisation will be the practice, and randomisation will be by centralised computer allocation.

### Sample size

For a total sample size of 228 practices, an alpha of 0.05, and a standard deviation of 7 prescriptions per 100, an intracluster correlation of 0.01 and a cluster size of 3 [7], the trial has 80% power to detect a minimum absolute mean difference of 1.5 prescribed item per 100 between intervention and control. The sample size was calculated using Stata 16's [16] command clustersampsi [17].

### Data collection

#### Data linkage and processing (prescribing outcomes)

Public Health Scotland holds the MIDAS and PRISMS databases. The MIDAS database contains claims information relating to all courses of NHS dental treatment provided by dentists in the General Dental Service since 1990. The PRISMS database contains information for all primary care prescriptions dispensed in community pharmacies (including dental prescriptions) since April 2004. The General Dental Practitioner NHS list number will be used as the single common identifier. The linked dataset will not contain any patient identifiable information and will be processed in accordance with the General Data Protection Regulations (GDPR) 2018. Updates of data will be received by the TRiaDS Office every month throughout the duration of the trial.

### Process measures data collection

Process outcomes will be collected via an online survey taken at the time of the in-practice training.

### Statistical analysis

The statistical analysis will be presented in detail in a pre-specified statistical analysis plan. The analysis will follow an intention-to-treat framework. The primary and secondary outcomes will be analysed at the dentist level. We will perform a multi-level analysis over a 1-year period, with time points of interest at 6- and 12-months post-randomisation and adjusting for the pre-intervention yearly prescribing rate (baseline prescribing rate) and practice size (single-handed/multi-handed). Fidelity, knowledge and confidence will be summarised by randomised arm using descriptive statistics.

### Subgroup analyses

Subgroup analyses on the primary outcome will explore the possible modification of treatment effect by practice size (single-handed/multi-handed) and pre-intervention levels of prescribing (high vs other prescribers; high is defined as the annual prescribing rate above the upper quartile). This will be done by including treatment-by-factor interactions in the model and they will be classified as exploratory analyses.

### Missing data

We do not anticipate any missing prescribing data in the analyses of the primary and secondary outcomes since data is collected routinely.

### Process evaluation

A theory-informed process evaluation will be conducted alongside the main trial to understand the mechanisms of impact and following best practice guidance [18]. The protocol for the process evaluation will be published separately.

### Interim analysis and data monitoring

This intervention is unlikely to have safety concerns and cross-over is not a concern. Given its low-risk, an independent monitoring committee will not be required and no interim analysis will be conducted.

### Monitoring and audit

The trial will be monitored to ensure that it is being conducted as per protocol, adhering to the UK Policy Framework for Health and Social Care Research, the principles of GCP and all other appropriate regulations. The approach to, and extent of, monitoring will be specified in a trial monitoring plan.

### Ethical considerations

The study interventions and data collection procedures involve minimal risk for dentists and dental practices and are unlikely to adversely affect their welfare. This is because practices have already voluntarily signed up for QIiPT in-practice IC training visits, QIiPT have been tasked with including a component on antibiotic prescribing within their IC training and are expected to evaluate the effects of their service delivery by NES. No NHS ethical approval is required as the only participants are healthcare professionals recruited by virtue of their professional role and GAfREC21 states that NHS ethical review is not required (confirmation of this was received from NHS Research & Development on 11 March 2020).

Consent will be sought from each dental practice gatekeeper to take part in the trial and show dental practice level data at the training session. Institutional ethical review was obtained on the 18 May 2020 by the University of Dundee Schools of Nursing & Health Sciences and Dentistry Research Ethics Committee (Reference: UOD\SDEN\2020\011_Clarkson).

### Amendments

Substantial and non-substantial amendments will be discussed with the Project Management Group (PMG), and when appropriate with the Research Methodology Group (RMG).

The amendment history will be documented in the protocol to enable the most recent protocol version to be identified. A current, up-to-date version of the protocol will be provided to all relevant members of the trial team and members of the PMG and RMG.

### Data protection

Participants will be reassured that all data which are collected during the course of the research will be kept strictly confidential. All personal data will be pseudonymised and processed in accordance with the General Data Protection Regulation Act 2018. The permission to access routine care data was approved by the Public Benefit and Privacy Panel for Health and Social Care on the 8th of October 2020 (Ref:1819-0207).

### Access to the final trial dataset

At the end of the trial, the trial statistician will have access to the full dataset to permit analysis. Requests for other access to the full dataset will be considered by the Project Management Group and the Sponsor. Statistical code for generating the results will be available.

### Organisation

#### The structure

The responsibilities for the administration and conduct of the study are reflective of the collaborative nature of the TRiaDS Programme as follows:

i) The financial administration of the study is the responsibility of the Dental Clinical Effectiveness Workstream office in Dundee Dental Education Centre (DDEC). Responsibilities include overall budgetary management, forecasting and authorisations, preparation of yearly budget reports and preparation of collaboration agreements with partner institutions.
ii) Day-to-day management of the study takes place in NHS Education for Scotland premises, primarily at the Dental Clinical Effectiveness Workstream office in DDEC. Responsibilities include generating and distributing individualised feedback materials, data collection and, in collaboration with the study team in the Health Services Research Unit (HSRU) in Aberdeen, data processing and data analysis.

The Dental Clinical Effectiveness Workstream office will be supported by staff in HSRU, University of Aberdeen, who will provide expert advice in respect of database development and management, the audit and feedback intervention and data analysis. In addition, HSRU are responsible for the randomisation of practices.

#### The project management group

The study is supervised by the Project Management Group (PMG). This consists of the budget holder and representatives from the study teams in DDEC (CS, JC, LY), HSRU (BG, ED, CR) and the NES dental offices in Glasgow (LM). Observers may be invited to attend at the discretion of the Project Management Group. Meetings will take place every 6 weeks on average.

#### The operations management group

Day-to-day responsibility for the study is the responsibility of the operations management group (OMG). The OMG comprises the PIs (BG, CS) and operational staff in DDEC (LY), and NES dental offices in Glasgow (LM). The groups will meet, on average, weekly at first and then fortnightly as the study progresses.

#### The research methodology group

The study will be overseen by the TRiaDS Research Methodology Group (RMG). This group will meet two times during the course of the study. Members of the host institution (NHS Education for Scotland) may also attend.

### Dissemination

The results of the study will be reported first to study collaborators. A summary of the findings will be sent to all participating practices and Health Boards, the Office of the Chief Dental Officer and the Dental Executive Team at NHS Education for Scotland.

It is anticipated that several peer-reviewed publications and conference presentations will result from the study. Decisions on authorship will be guided by the TRiaDS’s authorship policy.

## Discussion

The TiPTAP trial is an NHS Education for Scotland funded trial that presents an opportunity to embed robust evaluation into service delivery. It compares the effect of individualised audit and feedback with or without an in-practice training co-intervention. Even though we have evidence that audit and feedback works, there is uncertainty about how to maximise its effect [9]. Adding a co-intervention informed by barriers and facilitators to dental antibiotic prescription and including an action plan will allow us to address key questions in advancing the design of audit and feedback [19]. Specifically, whether audit and feedback will be more effective if it addresses and facilitators to behaviour change; and if it suggests clear action plans [20].

## Data Availability

Not applicable.
